# Zinc Oxide and Zinc Oxide Nanoparticles Impact on *In Vitro* Germination and Seedling Growth in *Allium cepa* L.

**DOI:** 10.3390/ma13122784

**Published:** 2020-06-19

**Authors:** Alicja Tymoszuk, Jacek Wojnarowicz

**Affiliations:** 1 Laboratory of Ornamental Plants and Vegetable Crops, Faculty of Agriculture and Biotechnology, UTP University of Science and Technology in Bydgoszcz, 6 Bernardyńska St., PL-85-029 Bydgoszcz, Poland; 2 Laboratory of Nanostructures, Institute of High Pressure Physics, Polish Academy of Science, 29/37 Sokolowska St., PL-01-142 Warsaw, Poland

**Keywords:** zinc oxide, nanoparticle, growth, micropropagation, onion

## Abstract

Zinc oxide nanoparticles (ZnO NPs) are ones of the most commonly manufactured nanomaterials worldwide. They can be used as a zinc fertilizer in agriculture to enhance yielding and to control the occurrence of diseases thanks to its broad antifungal and antibacterial action. The aim of this study was to investigate and compare the effects of ZnO submicron particles (ZnO SMPs) and ZnO NPs on the process of *in vitro* seed germination and seedling growth in onion (*Allium cepa* L. ‘Sochaczewska’), and to indicate the potential use of these compounds in onion production. In the experiment, disinfected seeds were inoculated on the modified Murashige and Skoog (MS) medium and poured with ZnO SMP or ZnO NP water suspension, at the concentrations of 50, 100, 200, 400, 800, 1600, and 3200 mg∙L^−1^. During three successive weeks, the germinating seeds were counted. Germination started most often on the second or third day of *in vitro* culture. The highest share of germination was recorded for seeds treated with 800 mg∙L^−1^ ZnO SMPs and ZnO NPs (52% and 56%, respectively). After the application of ZnO SMPs and ZnO NPs at the highest tested concentration (3200 mg∙L^−1^), the share of germinating seeds was only 19% and 11%, respectively. Interestingly, seedlings obtained from control seeds and seeds treated with ZnO SMPs and ZnO NPs did not differ statistically in terms of length, fresh weight, and dry weight of leaves, and roots. Both ZnO SMPs and ZnO NPs, in the concentration range from 50 to 1600 mg∙L^−1^, can be used to stimulate the germination process of onion seeds, without negative effects on the further growth and development of seedlings. There were no differences found between the action of ZnO NPs and ZnO SMPs, which suggested that the most important factor influencing seed germination was in fact the concentration of zinc ions, not the particle size.

## 1. Introduction

The global production of nanoparticles (NPs) has been increasing tremendously [1]. They are used in numerous disciplines, for example, optics, electronics, energy generation, materials science, medicine, and life sciences. In addition, they are released to the natural environment during production, transport, use, and disposal processes. Although plants are constantly exposed to naturally occurring NPs, exposure to engineered NPs is a new phenomenon and requires sufficient monitoring [2,3,4]. NPs are atomic or molecular aggregates that are characterised by unique physicochemical properties, such as high surface to volume ratio, high antimicrobial reactivity, high photocatalytic activity, and lower melting point, which were directly correlated with their small dimensions of less than 100 nm [5,6,7,8,9,10,11]. Due to their small size, chemical compounds in the form of NPs are absorbed by plants better than other, more traditional, bulk forms [12]. The interactions between NPs and plant cells, which cause both positive and negative morphological and physiological changes, strongly depend on the chemical composition, size, shape, surface covering, reactivity, concentration, and mode of NPs’ application, as well as plant genotype, age, and developmental stage [13,14,15,16,17,18,19,20]. In several plant species, various effects have been reported after NP application, such as stimulation or inhibition of seed germination/seedling growth [15,16], activation of genes involved in metabolism [17], induction of photosynthesis [18] or reactive oxygen species generation, and chromosomal aberrations [19]. NPs containing herbicides, fertilizers or nanoencapsulated nutrients, which are more effective on plants, are applied in agriculture and horticulture. Plant genetic engineers have also taken an interest in NPs. NPs can easily pass through the cell wall and release targeting genes to specific cellular organelles. Moreover, their application is not genotype specific and is more efficient than that of the currently known gene delivery systems, for example, biolistic techniques or *Agrobacterium* transformation [20,21].

Metallic zinc is an essential micronutrient in overall plant growth and development involved in a wide variety of enzymatic and physiological processes [22]. Zinc deficiency, occurring in the form of chlorosis and white necrotic spots on leaves, reduces growth and inhibits photosynthesis in many plant species [23]. The element acts either as a metal component of various enzymes or as a catalyst or structural cofactor and participates in protein, carbohydrate and nucleic acid synthesis, chlorophyll biosynthesis and energy production, as well as carbohydrate, protein, phosphate, and lipid metabolism [24]. Zinc increases the seed viability and radical growth in germinating seeds, affects the capacity for water uptake and transport, and reduces the adverse effects of heat, drought, or salt stresses. In addition, zinc plays an active role in the production of auxins and gibberellins [25,26]. A high concentration of zinc can damage cell functioning and disrupt a number of essential processes in a plant organism, due to the displacement of other elements that have a similar diameter and charge [27].

Zinc oxide nanoparticles (ZnO NPs) are among the most commonly manufactured NPs worldwide next to carbon nanotubes, gold, silver, and titanium dioxide NPs [28]. The annual output of ZnO NPs is between 550 and 5550 tons, which is 10–100 times higher than that of any other nanomaterial [4]. ZnO NPs are an inorganic compound appearing as a white powder and can be synthesized both by chemical methods (precipitation method, vapor transport method, and hydrothermal process) and biological methods using different plant extracts [5,29,30,31]. ZnO NPs are used in various products, such as gas sensors, biosensors, optical devices, solar cells, semiconductors, catalysts, paints, rubber products, ceramic sunscreen cosmetics, and drug delivery systems [2,3,4,5,30,31]. They are expected to be the ideal candidate for a Zn fertilizer in agriculture, enhancing crop productivity since the efficiency of both sulfate and chelate of Zn for soil and foliar application is low [32]. Moreover, ZnO NPs exhibit a broad antifungal and antibacterial action and could be applied to control the spread of and infections by a variety of plant pathogens [6,33,34]. Research and technologies for enhanced uptake and accumulation of micronutrients in edible plant parts could help solve the problem of people suffering from micronutrient deficiencies [32].

Only a few experiments have been performed to date to investigate the effect of ZnO NPs on plants. These experiments, most often, tested only the influence of ZnO NPs, and did not include a comparison with the results which could be obtained for ZnO applied at the same concentration. According to Siddiqiu et al. [20], ZnO NPs stimulate plant growth and development but the effects vary depending on the genotype. The toxic effect of ZnO NPs on plants shows a size-dependent characteristic, i.e., smaller particles are more toxic than the larger ones, and toxic effects increase in line with the increase in concentration [4,35]. In addition, the morphology of ZnO NPs affects their interactions with cell membranes, as well as their ability to penetrate into cells. The toxicity of rod-shaped particles is greater than that of spheres [36]. ZnO NPs at a concentration of 1.0 g·L^−1^ stimulates germination of soybean (*Glycine* Willd.) seeds under drought stress [26]. The application of 500 mg·L^−1^ ZnO NPs contributes to an increased protection from cadmium toxicity in maize (*Zea mays* L.) [37]. The results of the experiment conducted by Raskar and Laware [34] in onion (*Allium cepa* L.), on the one hand, showed that ZnO NPs at a lower concentration enhanced cell division, seed germination, and seedling growth. On the other hand, at a higher concentration, a decreased mitotic index, a decreased seed germination ratio, and an increase in frequency of chromosomal abnormalities were reported. However, as compared with our research, ZnO NPs were applied at a different concentration (10–40 mg·L^−1^) and the experiment was performed on filter paper, not on a plant micropropagation medium. The authors did not test simultaneously the effect of ZnO submicron particles (SMPs). Faizan et al. [3] found that ZnO NPs significantly increased growth and photosynthetic efficiency, as well as activated an antioxidant system in tomato (*Lycopersicon esculentum* L. ‘PKM-1’) seedlings. Plants of cluster bean (*Cyamopsis tetragonoloba* L.) treated with ZnO NPs showed a significant improvement in biomass, shoot and root length, root area, as well as chlorophyll content [29]. The application of Zn and ZnO NPs to the *in vitro* culture medium stimulated somatic embryo regeneration and shoot rooting in banana (*Musa paradisiacal* L.) plants [33].

Onion belongs to the Amaryllidaceae family and is an important vegetable crop in most parts of the world, consumed both in a raw or processed form. There is a stable increase in onion cultivation quantity and area, with the worldwide production above 93 M tons [38].

The aim of this study was to investigate and compare the effects of zinc oxide submicron particles (ZnO SMPs) and ZnO NPs at a wide range of tested concentrations (50, 100, 200, 400, 800, 1600, and 3200 mg∙L^−1^) on the process of *in vitro* seed germination and seedling growth in onion (*Allium cepa* L. ‘Sochaczewska’) and to indicate the potential use of these compounds in onion micropropagation.

## 2. Materials and Methods

### 2.1. Materials

The following reactants were used as received without further purification: zinc acetate dihydrate (Zn(CH_3_COO)_2_·2H_2_O, analytically pure, Chempur, Piekary Śląskie, Poland), ethylene glycol (C_2_H_4_(OH)_2_, analytically pure, Chempur, Piekary Śląskie, Poland), submicron particles of zinc oxide (ZnO SMPs, bulk form, pharmaceutical purity, production method by indirect (French) process, ZM SILESIA SA, Huta Oława, Oława, Poland) were used in the experiment. Seeds of *Allium cepa* L. ‘Sochaczewska’ were purchased at W. Legutko Przedsiębiorstwo Hodowlano-Nasienne Sp. z o.o. (Jutrosin, Poland).

### 2.2. Preparation of ZnO NPs

ZnO NPs were prepared using microwave solvothermal synthesis in accordance with our original procedure [39,40,41]. First, 36.65 g of Zn(CH_3_COO)_2_∙2H_2_O were dissolved in 550 mL of C_2_H_4_(OH)_2_ using a digital hot-plate magnetic stirrer (15 min, 70 °C, 450 rpm, SLR, SI Analytics, Mainz, Germany). The obtained clear solution of the precursor was decanted to a glass bottle (750 mL, Schott AG, Mainz, Germany), which was carefully closed by screwing on the cap and, subsequently, the bottle content was cooled to room temperature, and afterwards the water content analysis was carried out (H_2_O). A calculated mass of deionized water (2.6336 g) was added to the solution of Zn(CH_3_COO)_2_∙2H_2_O dissolved C_2_H_4_(OH)_2_ such that the final H_2_O content was 1.5 wt%, which was subsequently checked.

The reaction of solvothermal synthesis of ZnO NPs driven by microwave radiation was performed in a stop-flow microwave reactor, model MSS2 (270 mL, 12 min, 4 bar, 3 kW, 2.45 GHz, IHPP PAS (Warsaw, Poland), ITeE-PIB (Radom, Poland), ERTEC (Wroclaw, Poland)) [42]. The chemical reaction of synthesis of ZnO NPs is described as the following Equation (1) [40]:

(1) $$Zn{\left({\mathrm{CH}}_{3}\mathrm{COO}\right)}_{2}+2C_{2}H_{4}{\left(\mathrm{OH}\right)}_{2}\overset{C_{2}H_{4}{\left(\mathrm{OH}\right)}_{2},{\;H}_{2}O,\;T,\;P\;}{\to }{\mathrm{ZnO}}_{↓}+H_{2}O+2{\mathrm{CH}}_{3}{\mathrm{COOC}}_{2}H_{4}\mathrm{OH}$$

After the synthesis, the obtained post-reaction suspended matter of ZnO NPs was centrifuged (5000 rpm, MPW-350, MPW Med Instruments, Warsaw, Poland), subsequently, the supernatant was decanted, while the ZnO NPs were thoroughly rinsed with deionized water. The rinsing and centrifuging process was repeated 4 times. After the last process of centrifuging, a water suspension (≈3 wt%) was prepared from the obtained ZnO NP paste and frozen with the use of liquid nitrogen (N_2_) and dried (sublimation process) in a freeze dryer (Lyovac GT-2, SRK Systemtechnik GmbH, Riedstadt, Germany).

### 2.3. Characterization of ZnO Submicron Particles (SMPs) and ZnO NPs

The characterization of the ZnO SMPs and ZnO NPs, presented herein, was performed at an accredited research laboratory no. AB 1503 (Laboratory of Nanostructures, IHPP PAN, Warsaw, Poland), which operates in accordance with PN-EN ISO/IEC 17025:2018-02 [43]. The full description of the measurement procedures used is included in the publication [40,41].

X-ray powder diffraction (XRD) results were collected using the X-ray powder diffractometer (CuKα1) (23 °C, 20 from 10° to 100°, step of 0.02°, X’Pert PRO, Panalytical, Almelo, The Netherlands). On the basis of the XRD results, the average crystallite sizes were determined using Scherrer’s equation [39].

The crystallite diameter and size distribution were determined using the Nanopowder XRD Processor Demo web application [44,45].

The gas pycnometer (helium (He), (25 ± 1) °C, ISO 12154:2014, AccuPyc II 1340, V3.00, Micromeritics, Norcross, GA, USA) was used to determine the density of the skeleton (pycnometric density) of samples. The specific surface area of samples was determined by the Brunauer-Emmett-Teller (BET) adsorption isotherm equation using a surface area analyzer (nitrogen (N_2_), Gemini 2360, V 2.01, Micromeritics, Norcross, GA, USA) in accordance with ISO 9277:2010. Prior to measurement, samples were degassed for 2 h (150 °C, 0.05 mbar, VacPrep 061, Micromeritics, Norcross, GA, USA. On the basis of the determined specific surface area and density of the skeleton, the average size of particles defining their diameter was determined, with the assumption that all particles were spherical and identical [39].

The morphology of particles was determined using scanning electron microscopy (SEM) (ZEISS, ULTRA PLUS, Oberkochen, Germany).

The quantitative analysis of water in the precursor was carried out in accordance with the assumptions of the Karl Fischer method using the coulometric titration technique with the titrator (Cou-Lo AquaMAX KF, GR Scientific, Bedford, UK).

The average particle size in water suspensions was measured by the dynamic light scattering (DLS) method with the use of the Zetasizer Nano-ZS ZEN 3600 analyzer produced by Malvern Instruments Ltd. (Malvern, UK). The measurements were performed in accordance with ISO 22412:2008 with the following parameters: temperature 23 °C, water viscosity 0.9308 cP, temperature stabilization time in the measurement chamber 0 s, measurement angle 173° (backscattering), analysis model auto mode, and number of measurements 6. All tests were carried out in 12 mm disposable square polystyrene cuvettes (DTS0012). The sample temperature before measurement was stabilized in the laminar flow cabinet (S@feflow 1.2, BioAir S.p.A. Pero (MI), Italy) at room temperature (23) for 10 min. The samples were “delicately stirred” before placing in the analyzer to avoid the formation of air bubbles. Zetasizer 7.12, Malvern Instruments Ltd. (Malvern, UK), software was used. Samples of suspensions with the ZnO particle concentration of 100 ppm were selected for the tests.

### 2.4. Seed Preparation and Germination Experiment Parameters

After rinsing in running tap water, the seeds were incubated for 5 min in a 5% (*v/v*) detergent solution. Then, seeds were immersed in 70% (*v/v*) ethanol solution (1 min) and next in NaOCl (1.5% (*v/v*), 10 min). Subsequently, the seeds were rinsed twice, for 5 min, in sterile bi-distilled water. The seeds were inoculated on the modified Murashige and Skoog [46] medium with 660 mg∙L^−1^ CaCl_2_·2H_2_O, 41.7 mg∙L^−1^ FeSO_4_·7H_2_O, 55.8 mg∙L^−1^ Na_2_EDTA·2H_2_O, 30 g∙L^−1^ sucrose, and 8.0 g∙L^−1^ Plant Propagation LAB-AGAR^TM^ (BIOCORP, Warsaw, Poland). The pH of the medium was adjusted to 5.8, prior to sterilization (121 °C, 20 min). Then, for *in vitro* cultures, 40 mL of the medium was poured into 350 mL glass jars. Five seeds were inoculated in each jar. Immediately after seed inoculation, 1 mL of ZnO SMP or ZnO NP water suspension, at a concentration of 50, 100, 200, 400, 800, 1600, and 3200 mg∙L^−1^, was poured with a sterile tip on the medium and gently mixed to cover the whole medium surface (patent application in the Patent Office of the Republic of Poland). The control seeds were poured with sterile bi-distilled water.

The prepared ZnO SMP and ZnO NP suspensions, before application on seeds, were placed for 30 min in the Elmasonic S80(H) Ultrasonic Cleaner (37 kHz of ultrasonic frequency, of 150 W of effective ultrasonic power) (Elma Schmidbauer GmbH, Singen, Germany) to achieve a better dispersion of particles.

The cultures were kept in the growth chamber at (23 ± 1) °C, under 16/8-h (day/night) photoperiod, at approximately 35 μmol∙m^−2^∙s^−1^ of photosynthetic photon flux density (PPFD) provided by Philips TLD 36W/54 fluorescent lamps emitting cool daylight. In the first week of the experiment, the jars were covered with aluminium foil to limit the access of light.

Observations were performed daily for three consecutive weeks and based on the observations charts the dynamics of seed germination were prepared and the share of germinating seeds were determined. Next, biometrical data of seedlings were collected as mean values of leaf length (cm), fresh weight of leaves (mg), dry weight of leaves (mg), root length (cm), fresh weight of roots (mg), and dry weight of roots (mg).

The experiment was set up in a completely randomized design. For experimental treatment, 20 repetitions were used with 5 seeds each (1 jar). Data were statistically verified by applying Statistica 13.0 (StatSoft, Poland) software. The analysis of variance was performed. The means were verified with the Fisher test (*p* ≤ 0.05).

## 3. Results and Discussion

### 3.1. Morphology

**Figure 1:**
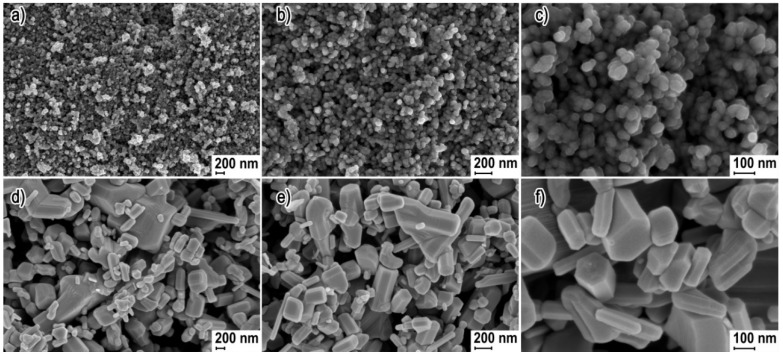
Scanning electron microscopy (SEM) images of zinc oxide nanoparticles (ZnO NPs) (**a**,**b**,**c**) and Zinc oxide submicron particles (ZnO SMPs) (**d**,**e**,**f**).

The scanning electron microscopy (SEM) images in Figure 1 present representative results of the morphology of ZnO NP and ZnO SMP samples. The ZnO NP sample was characterized by homogeneity of shape and size (Figure 1a–c). The size of the obtained spherical ZnO NPs ranged from ≅20 nm to ≅60 nm. The ZnO SMPs sample was characterized by heterogeneity in terms of shape and size (Figure 1d–f). ZnO SMPs with the shape of, among others, rods, bodies with a hexagonal base, or irregular bodies ranged from ≈100 nm to ≈2000 nm taking into account their largest dimension (length). The SEM images in Figure 1d,e show that the ZnO SMP sample was composed, among others, of irregular aggregates of sintered ZnO particles with the size of circa 2000 nm.

### 3.2. Phase Composition

**Figure 2:**
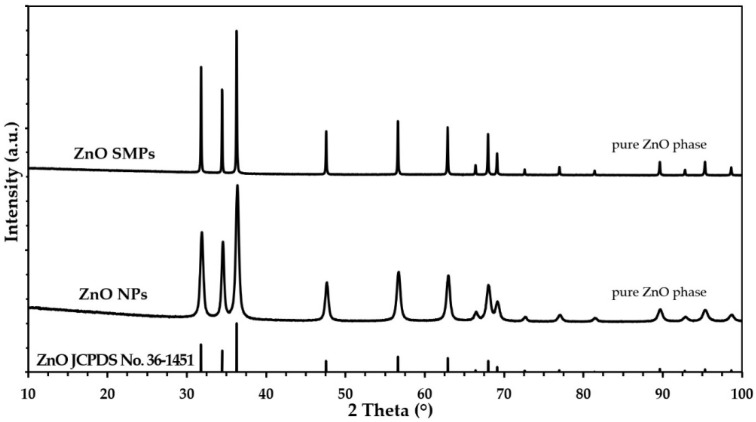
The X-ray powder diffraction (XRD) patterns for ZnO NPs and ZnO SMPs.

Figure 2 presents powder diffraction patterns, where all diffraction peaks were attributed to the hexagonal phase ZnO with the wurtzite structure, which has been reported in the literature for the reference material (JCPDS card no. 36-1451). The XRD results proved that the samples contained only crystalline ZnO and no crystalline impurities were detected that could fall within the detection range of the XRD method. A visual comparison of the obtained XRD results with one another (Figure 2), shows that based on the differences in the width of diffraction peaks, the ZnO SMPs sample was characterized by a considerably larger crystallite size as compared with the ZnO NP sample. The general rule when analyzing XRD results of samples of the same type of material is that the wider the diffraction peaks, the lower the crystallite size in the sample.

### 3.3. Density, Specific Surface Area, and Average Size and Crystallite Size Distribution

**Figure 3:**
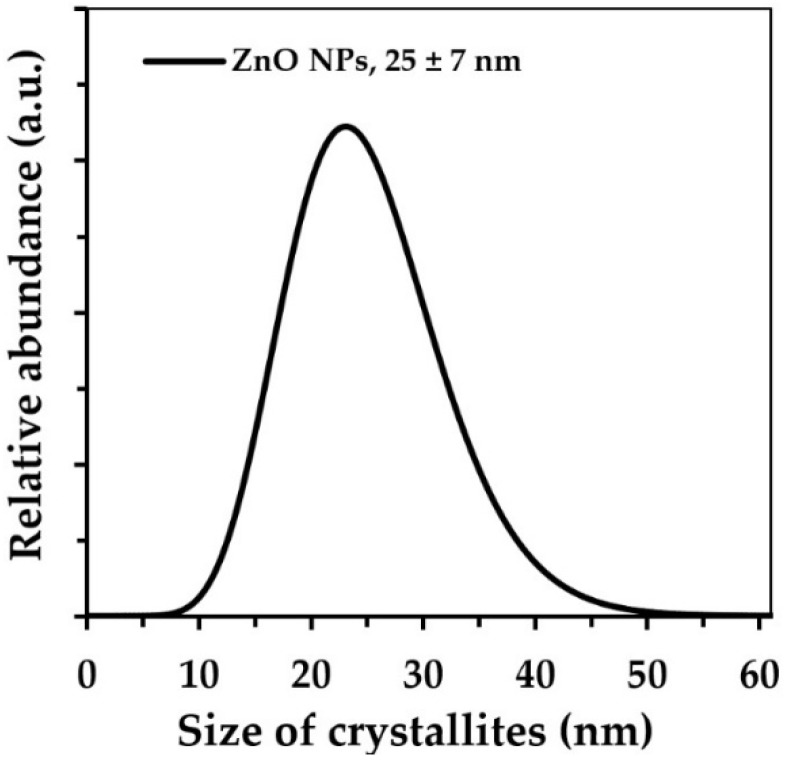
Crystallite size distributions of ZnO NPs.

The theoretical density of crystalline ZnO with the wurtzite structure is 5.61 g·cm^−3^ [39]. The achieved skeletal density and specific surface area for the ZnO NP sample were 5.24 g·cm^−3^ and 38.8 m^2^·g^−1^, respectively, whereas for the ZnO SMPs sample it was 5.59 g·cm^−3^ and 4.5 m^2^·g^−1^, respectively (Table 1). For nanomaterials (among others ZnO [39,40,47], hydroxyapatite [48], and zirconium dioxide [49]), a general correlation was noticed between the changes in the values of the specific surface area and density, i.e., a simultaneous impact on the change in the NPs’ size on the density and specific surface area. The causes of this correlation for nano ZnO were discussed in detail in our earlier publication [39]. However, the increase in the number of crystal lattice defects, which at the same time accompanies the increase in the specific surface area of materials, is regarded as the main cause of this correlation. The average particle size which was calculated based on the density and specific surface area results was 30 nm for the ZnO NP sample and 240 nm for the ZnO SMPs sample. A comparison of the crystallite size (Table 1, Figure 3), which was calculated by two methods, with the average size of single particles, showed a very good coincidence of results is visible, and therefore it can be stated that the particles in the ZnO NP sample were built of single crystallites (monocrystallinity). For the case of the ZnO SMPs sample, in turn, the average particle size (240 nm) was circa twice as big as the average crystallite size, which meant that some particles were built of several crystallites (polycrystallinity). The calculated crystallite sizes for the ZnO SMPs sample were burdened with a high error arising from the resolution of the diffractometer, namely the obtained peak width, and in the case of the ZnO SMPs sample, was at the threshold of the diffractometer resolution (Figure 2). The crystallite size distribution of the ZnO NP sample, presented in Figure 3, ranged from ≈15 nm to ≈55 nm and was consistent with the SEM results (Figure 1). The data obtained from the XRD analysis (peak width) for the ZnO SMPs did not permit calculating the crystallite size distribution, which indicated that the sample contained particles with the micron size, and this was confirmed by the SEM test (Figure 1).

### 3.4. Average Size and Size Distribution of Particles in Water Suspensions

**Figure 4:**
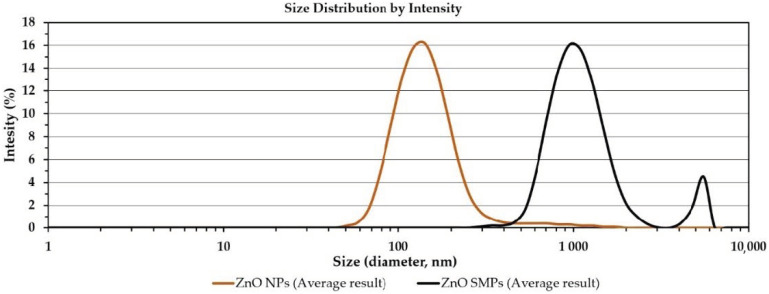
Particle size distributions of ZnO in suspensions with the concentration of 100 ppm (dynamic light scattering (DLS) method).

The summary of results of the average particle size analysis $\left({\bar{x}}_{\mathrm{DLS}}\right)$ in the suspension is included in Table 2 and in Figure 4. With respect to the DLS results, it must be borne in mind that the average particle size determined by this method refers to a sphere model (diameter) and is an average value referring simultaneously to the sizes of all particles, agglomerates, and aggregates present in the suspension sample during its analysis. The average particle sizes in the suspensions for the ZnO NP and the ZnO SMP samples were 174 and 1371 nm, respectively. Similar standard deviation values were achieved for both results. However, with regard to the comparison of the standard deviation values with the results to which they refer, it turned out that the standard deviation value was 29.9% of the value of the ${\bar{x}}_{\mathrm{DLS}}$ result for the ZnO NP sample, whereas it was merely 5.3% of the value of the ${\bar{x}}_{\mathrm{DLS}}$ result for the ZnO SMPs sample. In addition, a high standard deviation value was visible for the polydispersity index of the ZnO NP sample, which was 0.061 and accounted for as much as 22.7% of the value of the polydispersity index result. One more interesting correlation was observed. When the average particle size results in the suspensions (${\bar{x}}_{\mathrm{DLS}}$) were divided by the average particle size calculated for a single particle ($d_{SSA}$), the following results were achieved: 5.8 for the ZnO NP sample and 5.7 for the ZnO SMPs sample. These results could be associated with the achieved degree of particle agglomeration and with the achieved efficiency of the homogenization process in the ultrasonic cleaner.

The particle size distribution in the ZnO NP suspension ranged from ≈40 to ≈2000 nm, whereas for the ZnO suspension sample it ranged from ≈300 to ≈6500 nm. Figure 4 shows the characteristic “tail” of the distribution in the particle size distributions of two samples. The “tail” is a residue of the dry fraction of agglomerates and aggregates of ZnO particles, which was not dispersed during the homogenization with the use of the ultrasonic cleaner or which underwent secondary agglomeration.

### 3.5. Seed Germination and Seedling Growth

**Figure 5:**
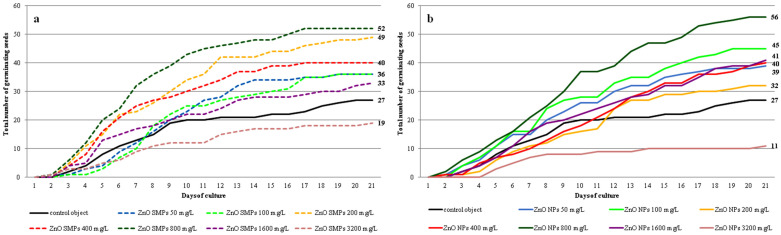
Dynamics of *Allium cepa* L. ‘Sochaczewska’ seed germination on the modified MS medium depending on ZnO SMPs (**a**) or ZnO NPs (**b**) treatment.

**Figure 6:**
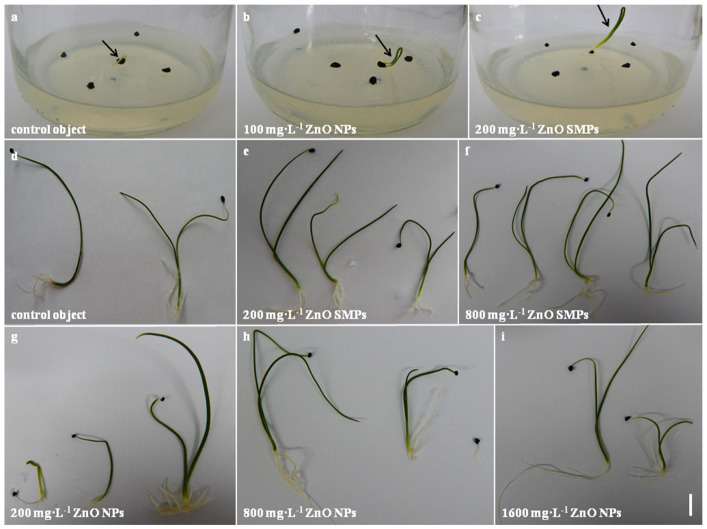
*In vitro* culture of *Allium cepa* L. ‘Sochaczewska’ on the modified MS medium (indicated with an arrow). First germinating seed (**a**); cotyledon developing (**b**,**c**); three-week-old plantlets obtained after application of ZnO SMPs or ZnO NPs at different concentrations (**d**–**i**). Bar = 1 cm.

Seed germination was observed in all experimental objects but with a different frequency (Figure 5a,b and Figure 6a–c). Most seeds treated with ZnO SMPs, irrespective of the concentration used, germinated between the third and the 12th day of culture. The germination of the ZnO NP-treated seeds did not proceed as fast during this period but was more intensive during the last days of culture. Germination started most often on the second day of culture (in the case of 50 mg∙L^−1^ ZnO SMPs and ZnO NPs, 200 and 400 mg∙L^−1^ ZnO NPs, 800 mg∙L^−1^ ZnO SMPs and ZnO NPs, and 3200 mg∙L^−1^ ZnO SMPs) or on the third day of culture (in the case of the control object, 100 mg∙L^−1^ ZnO SMPs and ZnO NPs, 200 and 400 mg∙L^−1^ ZnO SMPs, and 1600 mg∙L^−1^ ZnO SMPs and ZnO NPs). Germinating seeds on the medium with the addition of 3200 mg∙L^−1^ ZnO NPs were observed later, on the fifth day, and the intensity of germination in this treatment was the lowest. The dynamics of seed germination were most intensive on the media with ZnO SMPs and ZnO NPs at the concentration of 800 mg∙L^−1^ and on the media with 200 mg∙L^−1^ ZnO SMPs, as well as 100 mg∙L^−1^ ZnO NPs.

**Figure 7:**
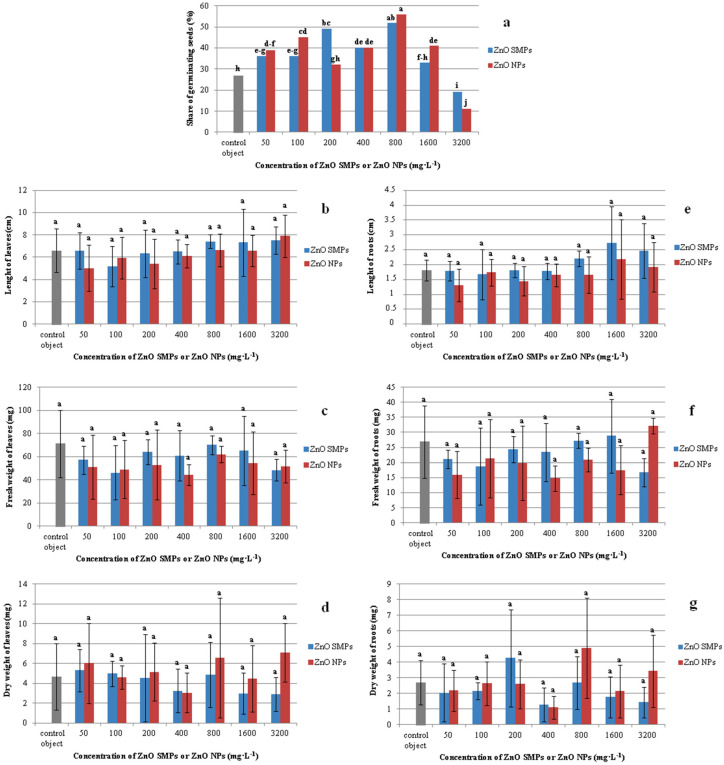
Share of germinating seeds (**a**) and seedling traits (**b**–**g**) in *Allium cepa* L. ‘Sochaczewska’ depending on ZnO SMP or ZnO NP treatments. Values represent percentage (**a**) or mean values ± standard deviation (**b**–**g**), 20 culture jars with five replications (seeds) were included in each treatment. Means on graphs followed by the same letter do not differ significantly at *p* ≤ 0.05 (Fisher test).

The use of ZnO SMPs and ZnO NPs in the concentration range from 50 to 1600 mg∙L^−1^ had a positive influence on seed germination (Figure 7a). The highest share of germination was observed in seeds treated with 800 mg∙L^−1^ ZnO SMPs and ZnO NPs (52 and 56%, respectively). These values were almost two times higher than in the control seeds (27%). The lowest share of germinating seeds was noticed after the application of ZnO SMPs and ZnO NPs at the highest concentration of 3200 mg∙L^−1^ (19 and 11%, respectively).

It can be assumed that only high concentrations, above 1600 mg∙L^−1^ of ZnO SMPs and ZnO NPs, are toxic for seeds of the tested onion cultivar. Raskar and Laware [34] investigated the influence of ZnO NPs with the size of 20 nm and at concentrations of 0, 10, 20, 30, and 40 mg·L^−1^ on seed germination in onion (*Allium cepa* L.). The ZnO NPs promoted seed germination at lower concentrations but reduced it at higher concentrations. At the highest concentration (40 mg·L^−1^), only 78.82% of seeds germinated and the maximum share of germination was observed at 20 mg·L^−1^ (96.52%), whereas the control seeds showed 94.28% seed germination. ZnO NPs applied at the concentrations of 250, 500, 750, and 1000 mg·L^−1^ inhibited tomato (*Lycopersicon esculentum* L.) seed germination, but the inhibition was not as high as in the case of bulk ZnO or ZnSO_4_ [23]. However, peanut (*Arachis hypogaea* L.) seeds treated with 400, 1000, and 2000 mg·L^−1^ ZnO NPs germinated better than the control seeds and seeds treated with ZnSO_4_. Such enhancement was attributed to the higher precursor activity of zinc NPs in auxin production. These results could indicate that a high zinc concentration in seeds has an important physiological role during seed germination and early seedling growth [32]. Zinc is a constituent of an enzyme influencing the secretion of indole acetic acid (IAA), which is a phytohormone (auxin) which significantly regulates plant growth. By increasing the level of IAA, zinc gives a positive response in seed germination [50]. We assume that the better germination of onion seeds in our experiment was in fact observed as a result of the stimulating influence of zinc ions released from ZnO SMPs and ZnO NPs on the biosynthesis of auxin.

Interestingly, the seedlings obtained from the control seeds and seeds treated with ZnO SMPs and ZnO NPs in the whole range of the tested concentrations did not differ statistically in terms of length, fresh weight, and dry weight of leaves (Figure 6d–i and Figure 7b–d). The mean value of the control leaf length was 6.61 cm, whereas, for example, the leaves of seedlings treated with 3200 mg∙L^−1^ ZnO NPs were 7.90 cm long and the leaves of seedlings treated with 50 mg∙L^−1^ ZnO NPs were 5.03 cm long. Seedlings from the control object and the 800 mg∙L^−1^ ZnO SMPs treatment were characterized by a high fresh weight of leaves, i.e., 71.08 and 70.12 mg, respectively, and at the same time, this value for the 400 mg∙L^−1^ ZnO NP treatment was only 44.25 mg. The dry weight of leaves of the control seedlings was 4.68 mg, whereas for the treatment with 3200 mg∙L^−1^ ZnO NPs it was 7.11 mg. The influence of ZnO SMPs and ZnO NPs on the root parameters was also nonsignificant. Seedlings produced from seeds after the application of ZnO SMPs at the concentration of 800, 1600, and 3200 mg∙L^−1^ had long roots, i.e., 2.21, 2.73 and 2.46 cm, respectively; and, in comparison, the roots of the control seedlings were 1.81 cm long (Figure 7e–g). The fresh and dry weights of roots varied from 32.15/3.43 mg for the treatment with 3200 mg∙L^−1^ ZnO NPs to 14.75/1.10 mg for the treatment with 400 mg∙L^−1^ ZnO NPs. Nevertheless, all these values did not differ statistically. In contrast, lower concentrations (10 and 20 mg·L^−1^) of ZnO NPs showed a significant enhancement in shoot and root lengths of onion seedlings, but higher concentrations (30 and 40 mg·L^−1^) showed decreased shoot and root lengths. A similar trend was observed in the fresh and dry weights of seedlings [34]. Tomato (*Lycopersicon esculentum* L.) plants with the roots exposed to ZnO NPs at the concentrations of 2, 4, 8 or 16 mg·L^−1^ for 15, 30, and 45 min showed an increase in length, as well as fresh and dry weights of roots and shoots. The maximum increase in these growth parameters was reported in the plants exposed to 8 mg·L^−1^ ZnO NPs for 30 min; values for shoot length (35.8%), root length (28.6%), shoot fresh and dry weights (21.9 and 27.6%, respectively), and root fresh and dry weights (19.9 and 27.7%, respectively) were higher than in the control plants [3]. Mahajan et al. [35] observed in mung (*Vigna radiata* (L.) R. Wilczek) that an increase in the ZnO NP concentration resulted in an increase in root and shoot elongation. Nevertheless, after exceeding a certain concentration, the root and shoot growth declined. Seedlings presented the best growth response for root (42.03% over the control) and shoot (97.87% over the control) after the application of 20 mg·L^−1^ ZnO NPs. At the highest concentration (2000 mg·L^−1^), the retardation in root (93.28% over the control) and shoot (14.85% over the control) lengths was observed. According to the authors, the growth reduction at higher ZnO NP concentrations could be attributed to damage to the basic architecture of roots (disruption of epidermis, cortex, and vascular cylinder). In the experiment conducted by Torabian et al. [12], the foliar spray of ZnO NPs, at a concentration of 2 g·L^−1^, improved the growth of sunflower (*Helianthus annuus* L.) plants by 19% measured as the dry weight of shoot as compared with the control plants that were not sprayed. As for the bulk ZnO form, the increase was lower and amounted to 7%. According to the authors, the mobility of NPs was very high in a plant, which led to a rapid transport of the nutrient to all plant parts and this factor could be responsible for a higher shoot dry weight of sunflower in ZnO NP treatment as compared with standard ZnO SMPs. Zinc can probably increase membrane stability and cell elongation. Therefore, an increase in radical growth led to an increase in fresh and dry weights [26]. However, surprisingly, in our experiment the application of ZnO SMPs and ZnO NPs at a wide range of tested concentrations, from low (50 mg·L^−1^) to very high (3200 mg·L^−1^), did not influence the length, or fresh and dry weights of three-week-old onion plantlets obtained *in vitro*. Such differences observed in the plant reaction to ZnO bulk form and ZnO NP application could result from plant genotype, plant part/organ (treatment of seeds, roots or leaves), and the environment of experiment (*in vitro*, *in vivo*), NP characteristics (size, shape, concentration, and type of stabilizing agent used), and/or exposure time [51].

We believe that the main reasons for the lack of differences in the obtained results in our experiment were the comparable chemical purity of the samples (ZnO SMPs and ZnO NPs), as well as the processes of particle agglomeration and sedimentation on the seed surface and on the medium surface. Particle agglomeration and sedimentation processes occurred for up to several hours after applying the suspensions to the seeds and were the decisive processes that cancelled out the “nano effect” in the direct contact of ZnO NPs with the seeds in the experiment that lasted for three weeks. The comparable chemical purity of the used ZnO samples (absence of impurities and absence of additives), in turn, led to the achievement of analogous effects of the impact of Zn^2+^ ions on the seeds from the point of view of thermodynamics. In other words, the different size of ZnO particles could affect only the speed of achievement of the equilibrium between the solid form of ZnO and “Zn^2+^” ions in the vicinity of the seeds but it could not contribute to the change in the final concentration of “Zn^2+^” ions, which resulted from the equilibrium constant (ZnO solubility product). Our findings are supported by Dimpka et al. [52], who suggested that although the initial metal solubility varied between NPs and bulk particles, the overall ion release levels were similar over time and both products (NPs and bulk particles) caused a similar accumulation of metals in plant organs.

## 4. Conclusions

The intensive development of nanotechnology has prompted undertaking research on the application of NPs in plant production. Nevertheless, the results of many experiments vary depending on NPs’ type, shape, concentration, and plant genotype. Our results confirm that both ZnO SMPs and ZnO NPs in the concentration range from 50 to 1600 mg∙L^−1^ can be used to stimulate the *in vitro* germination process of *Allium cepa* L.; ‘Sochaczewska’ seeds, without negative effects on the further growth and development of seedlings. The maximum response of germination was observed after the application of 800 mg∙L^−1^ ZnO SMPs and ZnO NPs. There were no toxic effects of ZnO NPs observed as compared with ZnO SMPs. Moreover, no differences between the action of ZnO NPs and ZnO SMPs were found, which confirmed the comparable chemical purity of the samples and suggested that the most important factor influencing seed germination was in fact the concentration of zinc ions, not the particle size. The different sizes of particles affected only the initial zinc solubility but did not contribute to the change in the final concentration of zinc ions and their influence on the tested onion seeds. Such promising results could be relevant for agricultural and horticultural practices related to stimulation of seed germination as well as plant micropropagation.

## 5. Patents

The elaborated method of *Allium cepa* L. seed treatment with the use of ZnO SMPs and ZnO NPs was sent to the Patent Office of the Republic of Poland as a patent application: “Method of stimulation of onion seed germination using zinc oxide or zinc oxide nanoparticles in water suspension (Polish title: Sposób stymulacji kiełkowania nasion cebuli z wykorzystaniem tlenku cynku lub nanocząstek tlenu cynku w suspensji wodnej)” (in Polish).

## Figures and Tables

**Table 1:** Characteristics of ZnO NPs and ZnO SMPs.

Sample Name	Skeleton Density, ρ_s_ ± σ (g·m^−3^)	Specific Surface Area, a_s_ (m^2^·g^−1^)	Average Particle Size from SSA, d ± σ (nm)	Average Crystallite Size, Scherrer’s Formula, d ± σ (nm)	Average Crystallite Size, Nanopowder XRD Processor Demo, d ± σ (nm)
ZnO NPs	5.24 ± 0.05	38.8	30 ± 2	27 ± 3	25 ± 7
ZnO SMPs	5.59 ± 0.03	4.5	240 ± 30	124 ± 11	-

SSA—Specific surface area, XRD—X-ray powder diffraction.

**Table 2:** Results of average particle size of ZnO in suspensions with the concentration of 100 ppm.

Suspension Name	Average Diameter, ${\bar{x}}_{\mathrm{DLS}}$ (nm)	Polydispersity Index, PI	Distribution Type	Average Size for Peak—Peak Intensity (nm %)
ZnO NPs	174 ± 52	0.269 ± 0.061	Monomodal	163.7–100
ZnO SMPs	1371 ± 73	0.380 ± 0.005	Bimodal	1082–93.1; 5247–6.9
